# Cesarean delivery in a pregnant woman with Ellis-van Creveld syndrome: a case report

**DOI:** 10.1186/s12884-026-09092-3

**Published:** 2026-04-17

**Authors:** Yuwen Yu, Wenming Zhuang

**Affiliations:** 1 Department of Obstetrics, Ningbo University Affiliated Women and Children’s Hospital, Ningbo, China; 2 Wenming Zhuang, Department of Obstetrics, Ningbo University Affiliated Women and Children’s Hospital, Ningbo, China

**Keywords:** Ellis-van Creveld syndrome, Cesarean delivery, Pregnancy, Rare diseases, Case report

## Abstract

**Background:**

Ellis-van Creveld (EVC) syndrome is a rare autosomal recessive disorder characterized by chondrodysplasia, postaxial polydactyly, congenital heart defects, and dental abnormalities. Reports of pregnancy in women with EVC syndrome are exceedingly uncommon.

**Case presentation:**

A 27-year-old woman with genetically confirmed EVC syndrome experienced an unplanned pregnancy complicated by fetal growth restriction and elevated umbilical artery Doppler indices. Through multidisciplinary team collaboration, she underwent cesarean delivery at 28 weeks, delivering a live male infant weighing 800 g. The mother had a favorable postpartum recovery. The neonate was admitted to the neonatal intensive care unit and was subsequently discharged in stable condition after 80 days of care.

**Conclusions:**

Pregnancy in women with EVC syndrome is rare and high-risk. Multidisciplinary management involving obstetrics, fetal medicine, critical care, neonatology, anesthesiology, and other specialties is essential to optimize maternal and neonatal outcomes.

**Supplementary Information:**

The online version contains supplementary material available at 10.1186/s12884-026-09092-3.

## Background

Ellis-van Creveld (EVC) syndrome, also known as chondroectodermal dysplasia, is an extremely rare autosomal recessive disorder with an estimated incidence of approximately 7 per 1,000,000 individuals [1]. It occurs more frequently in populations with a high rate of consanguinity, such as the Amish, Brazilian, Ashkenazi Jewish, and Arab communities [2–4]. The syndrome is characterized by ectodermal dysplasia (nail and dental abnormalities), chondrodysplasia, postaxial polydactyly, and congenital heart defects. Other organ involvement, including renal, hepatic, genitourinary, and central nervous system abnormalities, has also been reported [5–11]. Due to cardiopulmonary complications from congenital heart and thoracic malformations, over half of affected individuals die in infancy or early childhood [12]. Most reports describe pediatric cases, with few surviving into adulthood and even fewer pregnancies documented. Here, we describe a 27-year-old pregnant woman diagnosed with EVC syndrome who presented with fetal growth restriction (FGR) and elevated umbilical artery resistance and successfully delivered a live infant weighing 800 g at 28 weeks of gestation following multidisciplinary management.

## Case presentation

A 27-year-old gravida 3, para 1 woman at 27^+ 1^ weeks’ gestation was admitted for persistent FGR and elevated umbilical artery Doppler indices for 3 weeks. Her last menstrual period was March 9, 2025 (estimated delivery date, December 14, 2025). Early ultrasound confirmed gestational age. At 11^+ 6^ weeks, echocardiography showed a single atrium with a common atrioventricular valve and moderate-to-severe regurgitation. From 21 to 25 weeks, fetal growth remained below the 10th percentile, and Doppler studies indicated progressive umbilical artery flow resistance. At 27^+ 1^ weeks, amniocentesis with whole-exome sequencing was performed, further Doppler elevation (S/D 8.78) and low estimated fetal weight (797 g, 2nd percentile) prompted inpatient monitoring for risk of intrauterine demise.

Her obstetric history included a cesarean delivery at 7 months in 2021 for threatened preterm labor (neonate deceased at 3 months from accidental asphyxia) and a missed abortion at 2 months in 2024. She had no family history of hereditary disease.

**Fig. 1:**
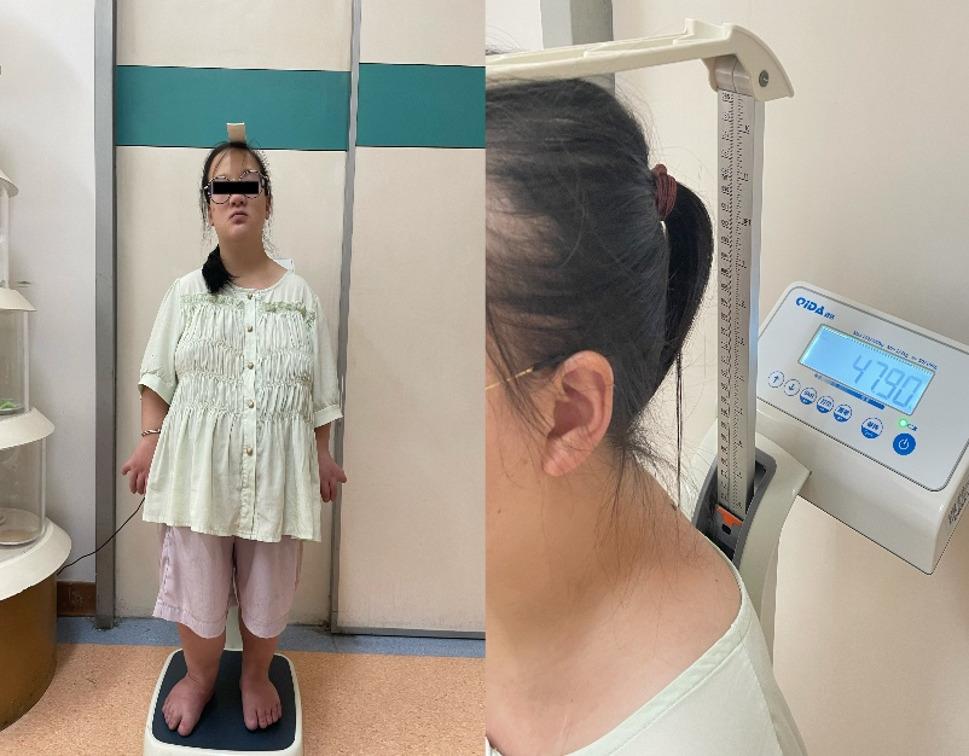
Profile of the patient showing short stature

**Fig. 2:**
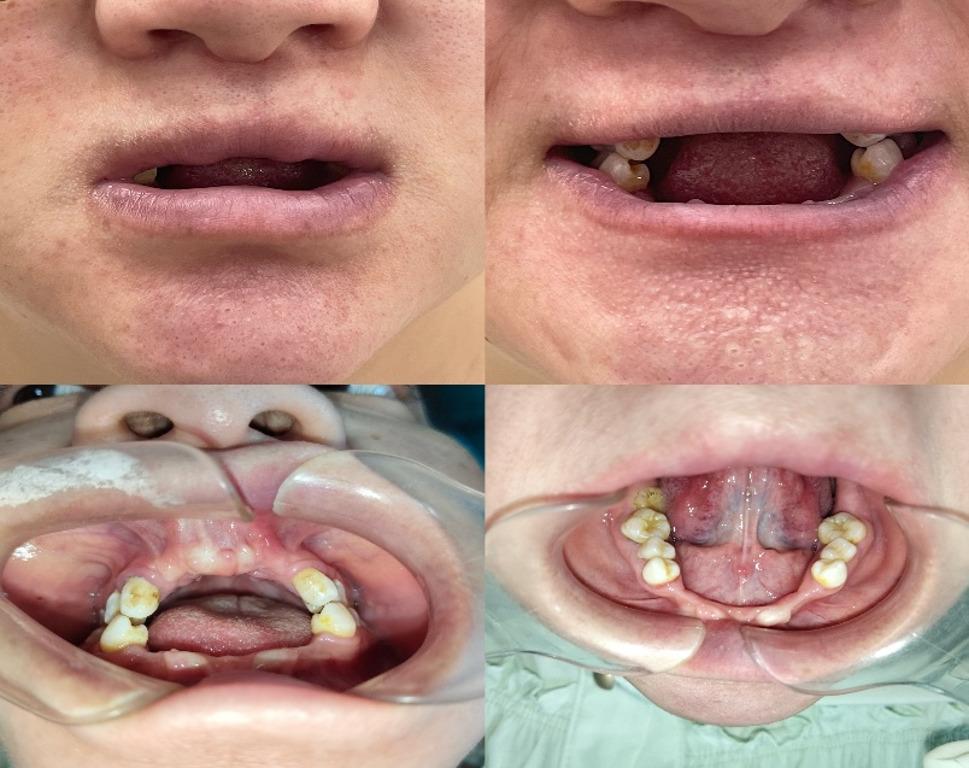
Oral malformations

**Fig. 3:**
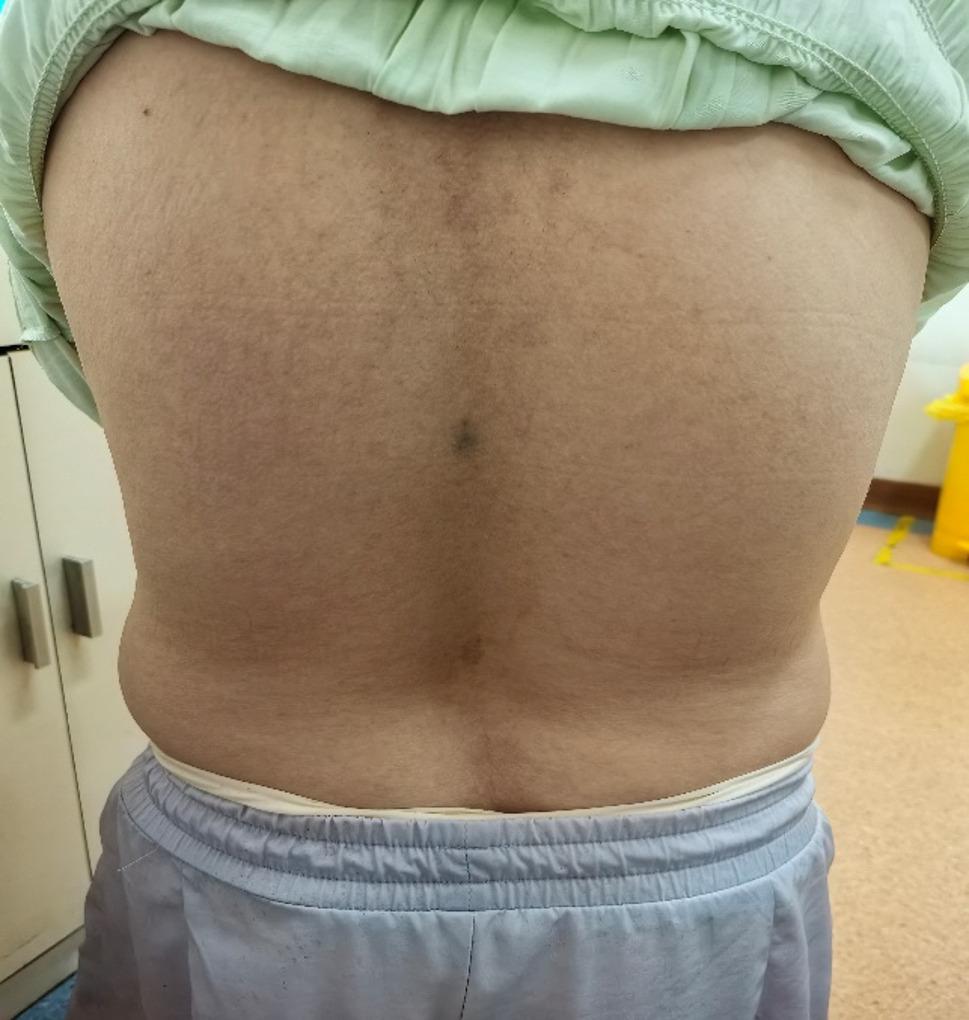
Spinal abnormality

**Fig. 4:**
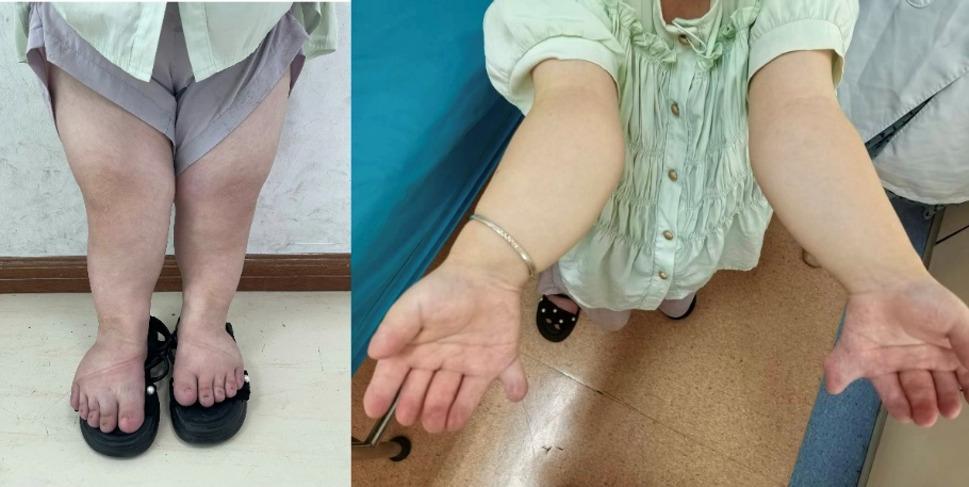
Unequal leg length and both elbows valgus

**Fig. 5:**
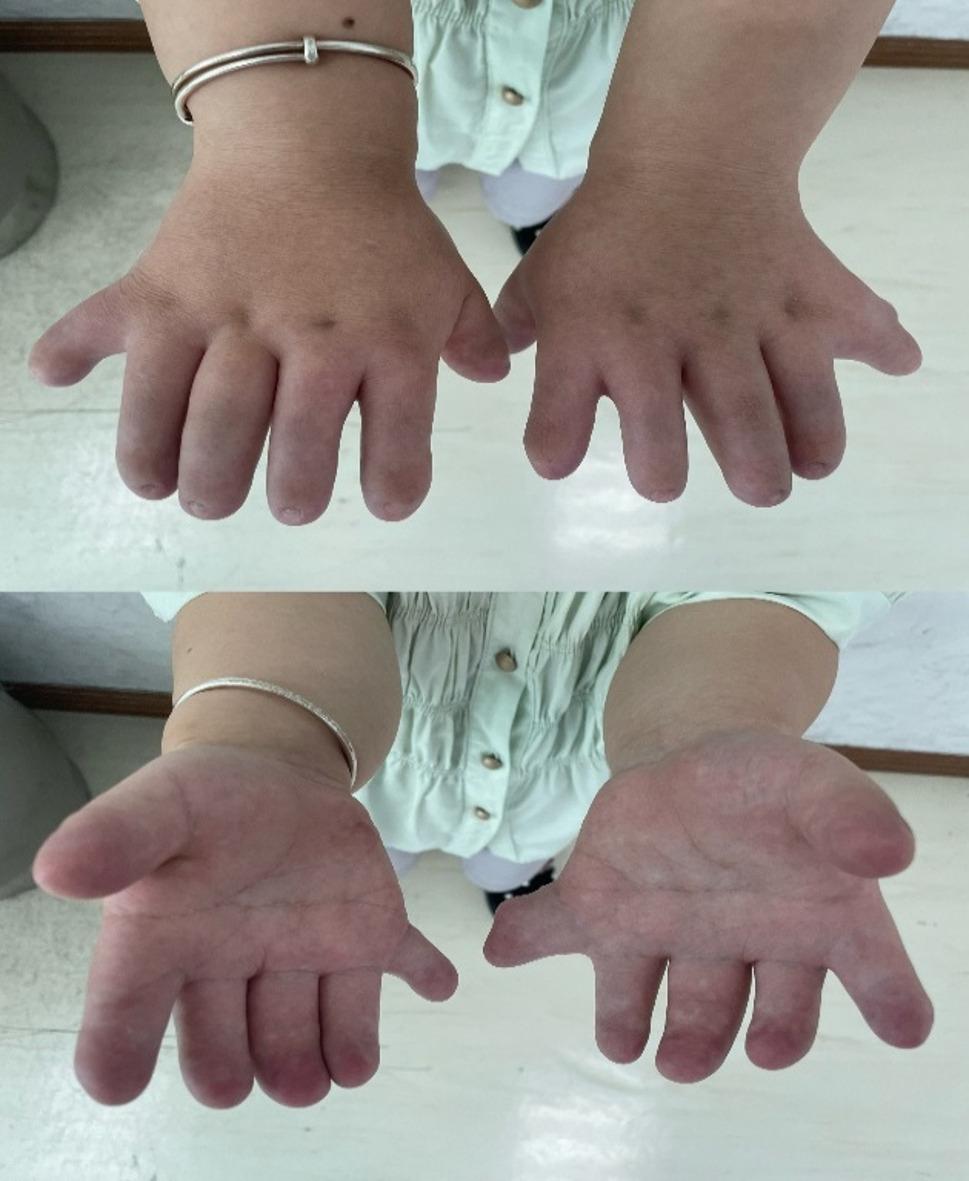
Post-axial polydactyly

On admission, vital signs were stable (SpO₂ 94–95%). She was 112.5 cm tall, weighing 47.9 kg (Fig. 1). Craniofacial features were largely normal, except for a slightly discontinuous upper lip vermilion border and missing anterior teeth (12–22, 33–43) with flattened alveolar ridges and a thickened upper labial frenulum (Fig. 2). Cardiac auscultation revealed an irregular rhythm with prominent systolic and diastolic murmurs. The spine had marked lumbar concavity but preserved mobility (Fig. 3). Both upper limbs were short, with bilateral elbow valgus (~ 45°) and postaxial hexadactyly of both hands, showing hypoplastic distal phalanges and nails. The lower limbs were short and slightly asymmetric, with flat feet and underdeveloped toenails (Figs. 4 and 5). No thoracic deformity or external genital abnormalities were observed. Electrocardiography showed sinus tachycardia with first-degree atrioventricular block, left axis deviation, and clockwise rotation. Echocardiography demonstrated a single atrium with a common atrioventricular valve and moderate-to-severe left-sided regurgitation. Other organ findings were normal.

Preliminary diagnosis: Suspected EVC syndrome, scarred uterus, FGR with elevated umbilical artery S/D ratio, G3P1 at 27 + 1 weeks.

**Fig. 6:**
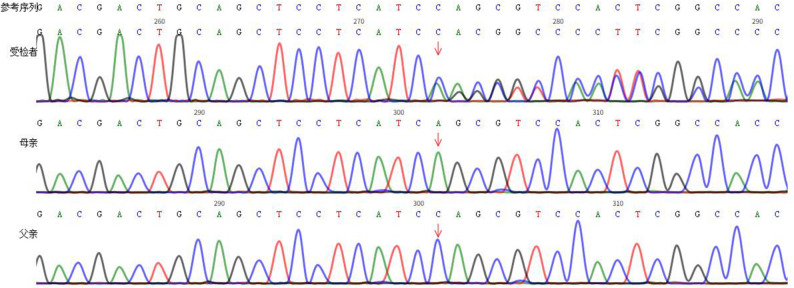
EVC gene c.528del site results of three family members

After admission, the patient received close fetal monitoring and antenatal corticosteroids to promote fetal lung maturation. At 27^+ 3^ weeks, ultrasound showed normalized umbilical artery flow, but at 28 weeks, repeat ultrasound revealed FGR with absent end-diastolic flow (AEDF). Considering fetal status and maternal symptoms, including nocturnal dyspnea and difficulty lying supine, informed consent was obtained for emergency cesarean delivery. The neonatal intensive care unit (NICU), anesthesia, operating room, and medical intensive care unit (MICU) teams were coordinated for perioperative care. Arterial and central venous lines were placed for invasive monitoring and fluid management. Due to single atrium, spinal deformity, and borderline oxygenation, general anesthesia with endotracheal intubation was chosen after airway and dental assessment. Fetal heart rate remained reassuring before induction, and cesarean delivery proceeded with full neonatal resuscitation.

An 800-g male infant in breech presentation with a nuchal cord was delivered via cesarean delivery. The amniotic fluid was mildly turbid (Grade I opalescence). The placenta measured 10 × 12 cm, with a 28-cm umbilical cord of 0.8 cm in diameter; the uterus and adnexa appeared normal. Post-delivery, slow Intravenous (IV) carbetocin (30 µg) and calcium gluconate (10 mL) were administered to promote uterine contraction. The patient was positioned with slight head elevation, and vasoactive agents (dobutamine and norepinephrine) were used to maintain hemodynamic stability; transient hypertension was managed with IV urapidil (10 mg).

The neonate exhibited no obvious congenital anomalies, with Apgar scores of 7, 9, and 9 at 1, 5, and 10 min, respectively, and was transferred to the NICU for further management.

Estimated blood loss was ~ 400 mL. Postoperatively, the patient was monitored in the MICU with ventilation, infection prophylaxis, and thromboprophylaxis. She developed transient pulmonary edema, which resolved with diuretics, and was successfully extubated on postoperative day 1, with face-mask oxygen at 5 L/min maintaining SpO₂ ~93%. Fever and elevated C-reactive protein were treated with antibiotic escalation (ceftriaxone to cefoperazone). Chest computed tomography revealed minor pulmonary exudates. Gastrointestinal recovery occurred by day 4, and inflammatory markers normalized by day 6. She was discharged in stable condition with an uneventful immediate follow-up. The neonate was discharged after 80 days in the NICU at a corrected gestational age of 39^+ 3^ weeks, weighing 2.38 kg. Telephone follow-up at more than four months postpartum indicated both mother and infant remained well.

Genetic findings: Amniocentesis identified a heterozygous EVC frameshift variant (c.528del) in the fetus and a homozygous variant in the mother, causing exon 4 loss of function (Fig. 6). The variant is pathogenic (American College of Medical Genetics and Genomics criteria) and extremely rare (gnomAD v2.1.1: 6.84 × 10⁻⁷ overall, 2.519 × 10⁻⁵ East Asian). A maternally inherited heterozygous titin (TTN) nonsense variant (c.57403 C > T, p.Gln19135Ter) was also detected, conferring potential risk for TTN-related cardiomyopathy.

## Discussion

EVC syndrome is a rare multisystem skeletal ciliopathy characterized by disproportionate short-limbed dwarfism, postaxial polydactyly, dental anomalies, and a high prevalence of congenital heart disease. Cardiac malformations, most commonly a single atrium with a common atrioventricular valve, occur in approximately 50–74% of affected individuals and represent the principal determinant of early mortality [7, 8, 13, 14]. EVC syndrome is most frequently caused by biallelic pathogenic variants in the EVC or EVC2 genes, which disrupt skeletal and cardiac development [9, 15]. However, genotype–phenotype correlations remain poorly defined, and clinical severity varies widely among affected individuals [15, 16, 17]. Importantly, pregnancy-related risk appears to be driven primarily by the extent of cardiopulmonary involvement rather than by the underlying genetic subtype. This phenotypic heterogeneity underscores the need for individualized risk assessment and multidisciplinary planning in pregnancies complicated by severe skeletal abnormalities and congenital heart disease.

The diagnosis of EVC syndrome in this patient was established based on characteristic clinical findings and confirmed by whole-exome sequencing, allowing differentiation from other skeletal ciliopathies [7, 12, 18]. Reports of pregnancy in women with EVC syndrome are exceedingly rare. To our knowledge, only one previously reported case described a favorable outcome following pre-pregnancy surgical correction of cardiac and limb defects [14]. In contrast, our patient had an uncorrected major cardiac lesion combined with extreme short stature, resulting in a markedly higher-risk obstetric profile.

In this case, the fetus demonstrated symmetrical growth restriction without structural anomalies, and genetic testing excluded EVC syndrome in the neonate. FGR was therefore most likely secondary to maternal cardiopulmonary compromise and placental insufficiency. Maternal cardiac dysfunction is known to impair uteroplacental perfusion and adversely affect fetal growth [19]. Consistent with this mechanism, antenatal Doppler studies revealed progressively abnormal umbilical artery flow, and intraoperative findings of a small placenta and thin umbilical cord further supported placental insufficiency as the primary contributor. The decision to proceed with delivery at 28 weeks of gestation was driven by concurrent deterioration in maternal cardiopulmonary status—including nocturnal dyspnea, orthopnea, and borderline oxygen saturation—and the progression to absent end-diastolic flow in the umbilical artery, reflecting severe placental compromise and a high risk of adverse perinatal outcome [20]. Although urgent delivery was required in this case, enhanced fetal surveillance, including assessment of ductus venosus Doppler waveforms, may provide additional guidance for timing of delivery in less rapidly progressive cases of severe FGR [20].

Pregnancy management in women with EVC syndrome necessitates individualized, risk-adapted decision-making. In the present case, the combination of significant cardiopulmonary disease and markedly reduced thoracic dimensions substantially limited physiological reserve. Current guidelines for pregnancy with congenital heart disease emphasize pregnancy continuation when maternal status is stable; however, the escalating cardiopulmonary burden of advancing gestation presented a difficult-to-quantify risk [19]. The decision to pursue early delivery therefore reflected a balance between fetal prematurity and the anticipated risk of irreversible maternal decompensation. Mode of delivery represented another area where guideline-based approaches required modification. While vaginal delivery is often preferred in women with cardiac disease to minimize surgical stress, this option was considered high risk in our patient due to extreme pelvic narrowing, limited cardiopulmonary reserve, and the potential for prolonged labor [19]. Planned cesarean delivery was therefore favored as a controlled strategy to reduce unpredictable intrapartum stress. Importantly, this decision was made in the context of uncertainty, acknowledging that no delivery approach was without risk. Anesthetic planning was particularly challenging. Combined spinal–epidural anesthesia, commonly recommended to reduce hemodynamic fluctuations during cesarean delivery, was deemed unsuitable in this patient because of severe spinal deformity and concerns regarding unpredictable spread of local anesthetics. General anesthesia, although associated with greater cardiopulmonary stress, was selected as the more controllable option. Airway management was further complicated by dental abnormalities and craniofacial features typical of EVC syndrome, necessitating advanced airway assessment and the involvement of experienced anesthesiologists.

This case highlights that, in women with EVC syndrome complicated by severe cardiac disease and extreme short stature, pregnancy management often requires deviation from existing guidelines. Decisions are frequently made under conditions of uncertainty, with trade-offs between competing maternal and fetal risks. The key transferable lesson from this case is the importance of early multidisciplinary planning, explicit discussion of alternative strategies, and individualized risk tolerance when managing rare and complex pregnancies for which high-quality evidence is lacking. Key management considerations across the course of pregnancy are summarized in Table 1.

**Table 1:** Key considerations for pregnancy management in women with Ellis-van Creveld syndrome

Stage	Key risks	Recommended assessments	Management considerations
Preconception	Undiagnosed congenital heart disease; limited cardiopulmonary reserve; genetic transmission risk	Genetic counseling; echocardiography; ECG; exercise test [19]; pulmonary function assessment; baseline oxygen saturation	Individualized risk stratification based on cardiopulmonary status; partner genetic testing; discussion of pregnancy feasibility and alternatives
Antenatal	Progressive cardiac overload; hypoxemia due to restricted thoracic capacity [21]; FGR	Regular echocardiography and ECG; oxygen saturation monitoring; fetal growth and Doppler surveillance (umbilical artery ± ductus venosus)	Close multidisciplinary follow-up; early cardiology involvement; semi-recumbent positioning and supplemental oxygen if needed; anticipatory anesthetic planning [22]
Timing of delivery	Maternal decompensation; placental insufficiency; fetal hypoxia	Maternal symptom progression; cardiac function; umbilical artery Doppler; fetal surveillance	Lower threshold for expedited delivery when maternal or fetal deterioration occurs; balance risks of prematurity against maternal cardiopulmonary compromise
Intrapartum	Hemodynamic instability; difficult airway; anesthesia challenges	Airway evaluation; spinal anatomy assessment; invasive hemodynamic monitoring [19]	Planned cesarean delivery preferred in severe skeletal or cardiac disease; individualized anesthesia choice [22]; availability of experienced anesthesiology and ICU teams
Postpartum	Acute heart failure due to fluid shifts; thromboembolism; infection	Continuous cardiopulmonary monitoring; volume status assessment	MICU-level care when indicated; cautious fluid and uterotonic management [19, 22]; thromboprophylaxis and infection prevention [22]
Neonatal	Prematurity-related morbidity; genetic risk	Neonatal genetic testing if indicated; cardiopulmonary monitoring	NICU admission; supportive care; long-term follow-up

*Abbreviations*: *ECG* Electrocardiogram, *FGR* Fetal Growth Restriction, *ICU* Intensive Care Unit, *MICU* Medical Intensive Care Unit, *NICU* Neonatal Intensive Care Unit

## Conclusions

Pregnancy and delivery in women with EVC syndrome are complex and high-risk. Their management requires close multidisciplinary coordination among obstetrics, maternal–fetal medicine, cardiology, anesthesiology, intensive care, neonatology, orthopedics, and dentistry. This report describes a rare pregnancy complicated by severe congenital heart disease and extreme short stature, resulting in a balanced maternal and neonatal outcome under individualized management. Findings from this single case cannot be generalized to all patients with EVC syndrome, particularly those with more advanced cardiopulmonary compromise or skeletal involvement, and long-term maternal and neonatal outcomes remain incompletely characterized. Nevertheless, this case highlights important considerations in pregnancy management for women with EVC syndrome. These insights may contribute to the evolving clinical experience needed to inform the management of similarly rare and high-risk pregnancies.

## Supplementary Information

Supplementary Material 1.

Supplementary Material 2.

## Data Availability

The datasets generated and/or analysed during the current study are available in the ClinVar repository, under accession numbers SCV007518766 – SCV007518767 (see https://www.ncbi.nlm.nih.gov/clinvar/submitters/510441).

## Abbreviations

EVC: Ellis-van Creveld
FGR: Fetal Growth Restriction
AEDF: Absent End-diastolic Flow
NICU: Neonatal Intensive Care Unit
MICU: Medical Intensive Care Unit
IV: Intravenous
TTN: Titin
ECG: Electrocardiogram
