# Supplementary material for: Cesarean delivery in a pregnant woman with Ellis-van Creveld syndrome: a case report
Source: BMC Pregnancy Childbirth. 2026 Apr 17;26:578. doi: 10.1186/s12884-026-09092-3 (PMC13217981; doi:10.1186/s12884-026-09092-3)
Supplement: Supplementary file 2 — Supplementary Material 2. [file 12884_2026_9092_MOESM2_ESM.pdf]

ClinVar Submission Portal

Home Contact Help My profile

Your Organizations

New organization

Department of Obstetrics and Gynecology

Edit Organization | Edit Submitter Group | Edit Personnel | View/add assertion criteria files | Request deletion

Organization information

Institution	Women and Children's Hospital Affiliated to Ningbo University
Address	No. 339 Liuting Street, Haishu District, Ningbo, Zhejiang, China 315000
Status	✓ Processed
Organization ID	510441

Submissions Summary Information

Type	Submissions	Records
In-Process	1	0
Released	0	0

Feedback

Your submissions

Start a new submission

- GenBank
- Sequence Read Archive
- Genome
- TSA
- BioProject
- BioSample
- API
- dbGaP
- GTR
- ClinVar

2 submissions

Submission	Title	App	Group	Status	Updated
SUB16048810	SUB16048810	ClinVar File Submission	clinvar-department-of-obstetrics-and-gynecology-8	clinvar: **Queued**	04:08
SUB16035316	Department of Obstetrics and Gynecology	ClinVar Organization	clinvar-department-of-obstetrics-and-gynecology-8	✓ clinvar: **Processed**	Mar 08

Feedback

搜索 中
